# An intervention to improve safe firearm storage for adolescents presenting with suicide ideation or attempt in a pediatric emergency department

**DOI:** 10.1186/s40621-022-00399-1

**Published:** 2022-12-21

**Authors:** Ashley Cleary, Frannie Kaczor, Maisie Finnegan, John Schimek, Abby Egen-Schimek, Erin O’Donnell, Marlene Melzer-Lange

**Affiliations:** 1 Community Education and Outreach, Children’s Wisconsin, Milwaukee, 53214 USA; 2 Family Services, Children’s Wisconsin, Milwaukee, 53226 USA; 3 Children’s Medical Group, Children’s Wisconsin, Milwaukee, 53226 USA; 4 Department of Pediatrics, Medical College of Wisconsin, Milwaukee, 53226 USA

**Keywords:** Suicide, Firearms, Firearm lockboxes, Emergency department, Injury prevention

## Abstract

**Background:**

Firearm injuries are the second leading cause of death in American youth aged 15 to 24, and over half of these deaths are suicides. Self-harm deaths in Wisconsin among adolescents have increased by 34% since 2006. Each year, our pediatric emergency department (ED) staff care for over 1100 children and adolescents who present with suicidal ideation or self-harming behaviors. We implemented an ED-based program aimed at improving the education given to families on reducing self-directed violence and providing firearm storage devices to families with the goal of reducing access to lethal means.

**Program description:**

Our program takes place in the pediatric ED of an academic children’s hospital and seeks to assist families of all patients who present with suicidal ideation or suicide attempt (SI/SA). In collaboration with our social workers, we reviewed their processes for interviewing and counseling families of patients who present with SI/SA. Social workers previously used a hospital-wide patient and family education sheet for safety planning that included information about safely storing medications and community mental health services. We teamed with our hospital’s health literacy and education committees and revised the teaching sheet to include more in-depth information about safe firearm storage. For families who were interested, we developed a process to provide up to two firearm lockboxes equipped with a combination lock. Working with risk management, the parent injury prevention product liability form was updated to include firearm lockboxes.

**Conclusion:**

We implemented a safe firearm storage program including development of a patient and family education sheet and provision of firearm lockboxes to families. Next steps under consideration include providing lockboxes for safe medication storage and establishing a follow-up system to assess proper use of firearm lockboxes and family and social worker satisfaction.

## Background

Since 2014, suicide has been the second most common cause of death in children and adolescents, from 10 to 24 years of age, in the United States (Centers for Disease Control and Prevention 2022). Self-harm deaths have increased since 2006, with deaths among adolescents in Wisconsin increasing by 34% (Curtin 2020). Firearms play a significant role in these deaths, given that over forty percent of suicides are caused by firearms (Centers for Disease Control and Prevention 2022). Average gun ownership per Wisconsin household was forty-three percent between 2007 and 2016 (Rand Corporation 2022). Adolescents with suicidal ideation are just as likely to have an accessible firearm in their home as those without a firearm (Simonetti et al. 2015).

For children and adolescents at-risk for suicide, clinicians have the unique opportunity to provide families counseling on lethal means restriction. Lethal means restriction is the reduction in access to firearms or other lethal means such as medications or sharp objects to persons at-risk of suicide. Lethal means counseling for parents of at-risk children and adolescents has been recommended by several national physician groups, including the American Academy of Pediatrics and the American Medical Association (American College of Emergency Physicians 2019; Ginsburg 1998; American Academy of Pediatrics Committee on Injury and Poison Prevention 1992; Knox et al. 2005). In a quality improvement project, Runyan et al. (2016) found that parents of youth presenting to a pediatric psychiatric emergency department with concerns for suicidality were accepting of Counseling on Access to Lethal Means (CALM) and reported improved firearm storage. Miller et al., conducted the SAFETY Study, a multi-center emergency department (ED) intervention for parents of youth at-risk for suicide presenting to the ED. They found that twice as many parents reported improved firearm storage after receiving counseling on lethal means restriction of firearms and medications while in the ED (Runyan et al. 2016) Additional research studies demonstrate the efficacy of brief interventions in improving safe storage practices among youth and adult populations in clinical settings. (Miller et al. 2020; Barkin et al. 2008; Grossman et al. 2000; Albright and Burge 2003). However, in a survey of emergency medicine physicians, most reported that they had not received training on firearm safety, nor would they be a credible resource to discuss firearm safety (Price et al. 2013).

Given that firearms are a predominant method for suicides nationally and in our state and seeing that Children’s Wisconsin Emergency Department/Trauma Center has over 1100 patient visits per year for suicidal ideation or attempt (SI/SA), our goal was to provide safe firearm storage in homes of our adolescent patients with SI/SA.

## Program description and methods

### Setting

Emergency Department/Trauma Center (EDTC) at Children’s Wisconsin, a free-standing, urban, pediatric hospital with a Level 1 trauma center, with approximately 70,000 visits annually, 24-h social work coverage, and an associated injury prevention program that provides home safety products to EDTC families in conjunction with the social work program.

### Social workers

Every patient and family who presents to the ED for SI/SA receives a social work consult, twenty-four hours a day, seven days a week. Licensed social workers evaluate the patient utilizing the Columbia Suicide Severity Rating Scale (Posner et al. 2011), assess their safety, provide mental health resources, and assist with identifying either inpatient or outpatient mental and behavioral health treatment options.

### Program development

Hospital social workers, injury prevention specialists, pediatric residents, and a pediatric emergency medicine physician collaborated on the development of this program. While social workers had been counseling on lethal means restriction as part of discussions with families of at-risk patients, they were interested in developing a bundle that would include a patient/family education sheet for use in the EDTC and at discharge, and recommendations on safe firearm removal or storage, including provision of firearm lockboxes.

Social workers utilize a behavioral health template for the electronic health record where they document their lethal means restriction family education. A patient/family education sheet, specifically tailored for EDTC usage, was developed that included general information about behavioral health emergencies, considerations on lethal means restrictions (safe storage of medications, firearms), and telephone numbers for local and national suicide prevention resources. The patient/family education sheet was reviewed by our health literacy team to be at the sixth grade reading level in both English and Spanish. The patient/family education sheet is reviewed with the family at the time of the ED visit and interpreters are used for non-English speaking caregivers. In the case of caregivers who could not read, social workers review the information on the patient/family education sheet verbally.

While the social work team had previously advised families to remove all firearms completely from the home, it was noted that one barrier to safe firearm removal was the inability to find a place to move firearms out of the home, especially on short notice. Social workers noted that families were frequently reluctant to move their firearms to relatives’ homes or to a police station. Through funding from the American Academy of Pediatrics Community Access to Child Health program, we obtained firearm lockboxes for distribution to families at no cost. The lockboxes are accessible via a combination lock and can hold one handgun. We selected a small lockbox to make it feasible to keep in the home as it occupies minimal space. The lockbox does not need to be bolted to the floor or the wall, so it offers a safe storage option for families who live where housing cannot be modified.

Our injury prevention and social work teams revised a previously developed Home Safety Product Release of Liability form to include firearm lockboxes. Included on the form are checkboxes for a variety of safety products, a paragraph that notes that parents/caregivers have received the products and understand that they will not hold the hospital liable for any issues, and a place for the parents/caregivers to sign the document. A copy of the completed form is kept in the patient’s electronic health record and one is kept in the injury prevention office. The Children’s Wisconsin Risk Management Office reviewed and approved the form. Social workers were trained to ask families if they needed a firearm lockbox as part of their discussion about lethal means restrictions. They also received training to discuss the use of the firearm lockboxes, the new patient/family education sheet, and the release of liability form completed with families who received a firearm lockbox.

## Discussion

Creating the firearm lockbox program has led to increased education and awareness by social workers as to the importance of prevention and safe storage of firearms. Given the timely manner that these lockboxes are provided, families can put them into use immediately after a traumatizing event. If families were solely given education on safe storage, they would have to find the time and funds to purchase lockboxes themselves. This program eliminates the burden of research, time, and funding for families by supplying high-quality lockboxes at the time of their child’s ED visit.

This resource has been viewed by our social work team and seen as a necessary service to help prevent injuries from firearms. The availability of firearm lockboxes serves as a prompt for social workers to discuss and emphasize the importance of lethal means restriction with families. While our program was meant to address suicidal concerns, social workers also identified two other situations in which lockboxes could be distributed: (1) to families of patients sustaining either an unintentional or intentional gunshot wound. (2) to prospective foster families who were required to have their firearm locked so they could accept foster children.

One potential consequence of providing the lockbox is improper usage. Families may either not use the gun lockbox, leave it unlocked, give it to someone else, or lose it. To mitigate this consequence, education on the firearm lockbox and safe storage are given to the families when the item is distributed. Another shortcoming of the lockbox is that it stores only one handgun at a time. For families with more or larger firearms in the household, a larger firearm safe is recommended. To assist these families, the social workers provided education regarding general firearm safe storage so they can better select an appropriate storage device. While some community-based organizations addressing suicide prevention have designated locations to temporarily store firearms, such as gun dealers, self-storage companies, and shooting ranges, these may not be immediately accessible to families (Runyan et al. 2017; Colorado Firearm Safety Coalition 2022). Another potential barrier is having enough storage space in the ED to keep the firearm lockboxes. Our program keeps a small number of lockboxes in the ED and stores the remainder in the social work offices.

Next steps for our program include provision of medication lockboxes, formal assessment of social worker satisfaction with the program, and follow-up with families who received lockboxes to assess their lockbox use and satisfaction. We plan to offer lockboxes for homicidal patients and in ED cases where the need for safe firearm storage arises. Lethal means restriction counseling at the time of a patient’s ED visit gives caregivers an opportunity to prevent a tragic event. We believe providing a firearm lockbox at the time of the visit along with education on its use also provides the caregiver with a concrete safety device and reminder to reduce lethal means in their home. Families are in crisis when they have learned that their child has considered or attempted suicide. ED health providers have a crucial responsibility to assist patients and families with both medical care and injury prevention strategies to address future suicidal issues.

## Data Availability

Not Applicable.

## Abbreviations

ED: Emergency department
EDTC: Emergency department/trauma center
SA: Suicide attempt
SI: Suicidal ideation
